# Microwave head imaging systems for early brain tumor detection: antenna designs and emerging substrates

**DOI:** 10.1016/j.mex.2025.103726

**Published:** 2025-11-16

**Authors:** Jinu Mathew, Osamah Ibrahim Khalaf, Navin M George, Ancy Michel, Neethan Elizabeth Abraham, Deema Mohammed Alsekait, Sharf Alzu’bi, Diaa Salama AbdElminaam

**Affiliations:** a Department of Electronics and Communication Engineering, Karunya Institute of Technology and Sciences, Coimbatore, India; b Al-Nahrain University, Al-Nahrain Renewable Energy Research Center, Baghdad, Iraq; c Department of Electronics and Communication Engineering, Nehru Institute of Engineering and Technology, Coimbatore, India; d Department of Information Technology, College of Computer and Information Sciences, Princess Nourah bint Abdulrahman University, P.O. Box 84428, Riyadh 11671, Saudi Arabia; e Department of Information Technology, College of Engineering and Technology, Royal University for Women, West Riffa, Bahrain; f Faculty of computers and Artificial Intelligence, Benha University, Benha, Egypt; g Jadara Research Center, Jadara University, Irbid, 21110, Jordan

**Keywords:** Brain tumor, Microwave head imaging antenna, Substrate, Specific absorption rate (SAR), Gain, Reflection co-efficient, Artificial intelligence and machine learning

## Abstract

Early detection of brain tumors is essential for successful treatment and better patient outcomes. Traditional imaging methods like X-rays, MRIs, CT scans, and PET scans have been important in detecting brain tumors, but they are expensive with many drawbacks and areas where access is limited. Antenna-based method has recently emerged as a practical alternative for real-time, non-invasive detection of brain tumors. This paper explores different antennas and various types of substrates that are adaptable to human sensitive tissues for detecting brain tumors. This review highlights the antenna working principles, and the advantages and challenges associated with each type. The effectiveness of several antenna-based methods in medical diagnostics, including microwave imaging and ultra-wideband (UWB) systems, is discussed. To assess their impact on detection accuracy, essential factors such as penetration depth, resolution, operating frequency, and antenna design are considered. The integration of antennas with machine learning and signal processing techniques is investigated.

## Review Highlights

• Brain tumors are presented with emphasis on their types, diagnostic challenges, and the necessity of early detection.
• Antenna technology in medical imaging is reviewed with focus on its evolution, role in brain tumor detection, and associated advantages and limitations.
• Key antenna design considerations for brain tumor detection are outlined, including frequency selection, size and configuration, material choice, and signal processing integration.
• Future directions and challenges are highlighted, addressing microwave imaging antennas, ultra-wideband systems, RF coils for MRI, and other emerging technologies.

## Graphical abstract

**Figure:**
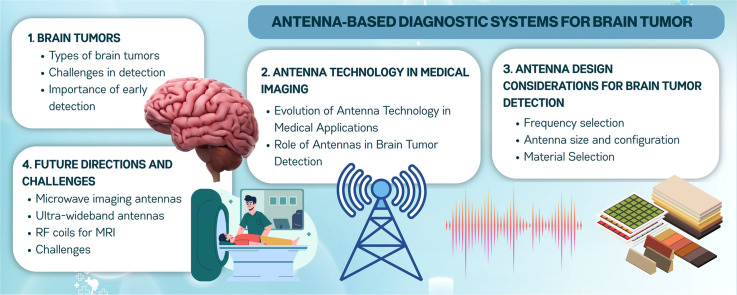

**Specifications table**

**Table:** 

Subject area	Engineering
**More specific subject area**	Antennas in medical imaging
**Name of the reviewed methodology**	Literature search, Data Inclusion and Exclusion, Data Extraction and Categorization, Analysis and Synthesis, Visualization and Representations. Methodology name: Systematic Literature review (SLR) – for scientific rigor and reproducibility.
**Keywords**	Brain tumor; Microwave head imaging antenna; Substrate; Specific Absorption Rate (SAR); Gain; Reflection co-efficient; Artificial Intelligence and Machine Learning
**Resource availability**	Hossain, M. M., Hasan, M. R., Rahman, M. A., Sarker, M. R., & Alam, M. S. Microwave brain imaging system to detect brain tumor using metamaterial loaded stacked antenna array. Sci. Rep. 12 (2022) 20,944. https://doi.org/10.1038/s41598–022–20944–8 Hamza, A. B., Sali, A., Bouaoune, A., & Elfergani, I. Design and experimental validation of a metamaterial-based sensor for microwave imaging in breast, lung, and brain cancer detection. Sci. Rep. 14 (2024) 67,103. https://doi.org/10.1038/s41598–024–67103–9 Hossain, M. M., Hasan, M. R., & Rahman, M. A. Brain tumor segmentation and classification from sensor-based microwave head imaging. Front. Oncol. 13 (2023) 10,046,629. https://doi.org/10.3389/fonc.2023.10046629 Wongkasem, N., Jitpraphai, S., & Rakluea, T. Multiple-point Multiple-Point Metamaterial-Inspired Microwave Sensors for Early-Stage Brain Tumor Diagnosis-inspired microwave sensors for meningioma tumor detection. Sensors 24 (2024) 5953. https://doi.org/10.3390/s24185953 Abbosh, Y. M., Sultan, K., Guo, L., & Abbosh, A. Non-uniform antenna array for enhanced medical microwave imaging. Sensors 25 (2025) 3174. https://doi.org/10.3390/s25103174
**Review question**	1. How has antenna technology evolved to support medical imaging, and what specific advantages does it offer for brain tumor detection compared to conventional imaging modalities? 2. What are the critical antenna design considerations—particularly frequency selection, size, material choice, and configuration—that determine the effectiveness of brain tumor detection systems? 3. In what ways do advanced signal processing techniques enhance the accuracy and resolution of antenna-based brain tumor imaging? 4. What are the major challenges—both technical and clinical—in translating antenna-based diagnostic systems from laboratory research to real-world clinical practice? 5. Which emerging antenna technologies, such as microwave imaging, ultra-wideband antennas, or RF coils for MRI, hold the greatest potential to improve future brain tumor diagnostics?

## Background

**Fig. 1:**
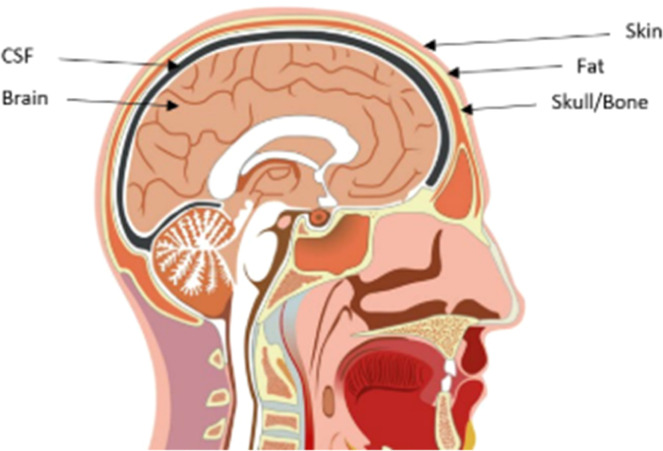
Cross sectional view of human head [1].

The brain is a complex structure that processes information from the senses, manages memories, emotions, and thoughts and controls our body's movements [1]. A cross-sectional view of human head brain is shown in Fig. 1. An abnormal mass of tissue that grows within the skull is known as a brain tumor. This condition can be diagnosed through medical head imaging and biopsy. Biopsy is associated with several disadvantages including low sensitivity, danger during biopsy process and a long waiting time for results. Head imaging helps to find and locate diseases associated with the brain [2,3].

Today, numerous imaging methods are used to detect anomalies in the human head such as computed tomography (CT) scans, magnetic resonance imaging (MRI), X-rays, ultrasounds or positron emission tomography (PET). The previously discussed technologies have several drawbacks, including ionizing radioactivity, dangerous radiation, increased costs, flexibility, and the impossibility of continuous monitoring [1,5]. Recently developed microwave brain imaging systems have been highly recommended due to their low cost, speed, non-invasiveness, and non-ionizing radiation usage [6,7].

Head imaging antennas are a quickly developed technique for imaging the brain and other neurological diseases. A comprehensive overview of antenna-based head imaging is discussed here. Recently, antenna-based imaging has become a viable option for detecting brain tumors [8]. This inexpensive method uses electromagnetic waves to scan the brain and identify tumors, which also helps in the speedy diagnosis and follow up [5]. Microwave imaging procedure is a contactless procedure, meaning that no physical contact or ionizing radiation is needed. Moreover, the quality of the images is improved through developed signal processing technology, thus providing reliable information for diagnosis to the clinicians.

## Method details

### Brain tumors: an overview

**Fig. 2:**
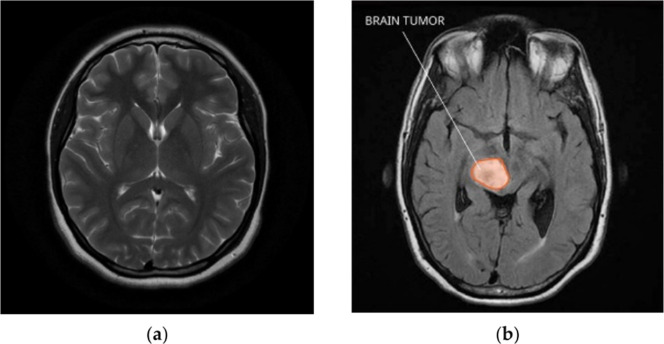
MRI images of brain with (a) No Tumor and (b) Tumor [9]**.**

The central nervous system or CNS is made up of the spinal cord and the brain [2]. One of the biggest and most complex organs, the brain regulates every bodily function as well as thought, memory, emotion, touch, motor skills, vision, respiration, temperature, and hunger [3]. A growth of abnormal cells in or around the brain is described as a tumor. Fig. 2 shows an MRI images of brain tissue affected with tumor and without tumor. One of the high-mortality-rate cancer diseases is brain tumor, which is ranked as fifth [4]. Brain imaging is the technique used to visualize and localize abnormal cells [5,6]. Brain tumors constitute the most common cancer among children aged 0–14. The highest incidence rate for brain tumors occurs among individuals aged 85 years and older. Among adolescents and young adults (ages 15–39), brain tumors stand as the third most common cancer and the third leading cause of cancer-related death within this age group [7,8].

### Types of brain tumors

The World Health Organization (WHO) has categorized several types of brain tumors based on the location of the origin, the kind of tissue involved, and whether they are malignant or benign, (primary or secondary) and additional contributing elements [3]3. Classification of a brain tumor is based on the growth rate, typical location, and size at the time of diagnosis, and who they affect. There are two types of brain tumors: primary and secondary [10]. Primary brain tumors are those that originate in the brain, while secondary brain cancers spread to the brain. Primary brain tumors can be cancerous (malignant) or non-cancerous (benign) [11]. Benign tumors are slow-growing tumors that are most common in individuals between the ages of 50 and 60. A malignant brain tumor is a cancerous growth in the brain and grows faster than benign tumors [12].

**Fig. 3:**
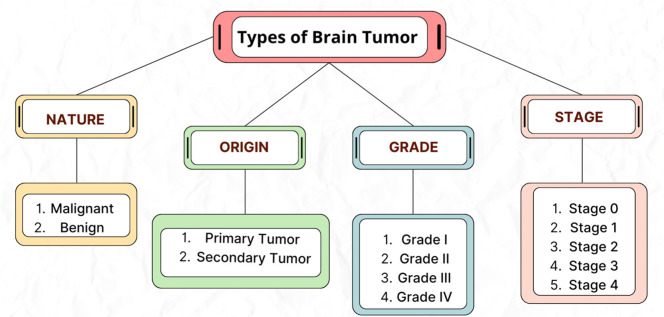
Type of brain tumors**.**

Fig. 3 shows a grading system is designed for classifying a tumor as either harmful or harmless, indicating how quickly the cells may expand or are likely to spread depending on its tissue appearance [13]. This data is essential for forecasting results and organizing the course of treatment. Less aggressive lower-grade tumors (grades I and II) are typically linked to long-term survival. Higher-grade tumors (grade III and IV) can do more harm, develop faster, and are frequently more challenging to treat [14,15].

To determine the extent of a spreading a tumor, the progression stage is often quantified using stages 0 through 4. Stage 0 refers to aberrant cancerous tumor cells that are unable to spread to nearby cells. On the other hand, malignant tissues grow quickly in stages 1–3 while they spread to different areas of the patient's body in stage 4 [13].

### Challenges in detection

In general, patients with cancer do not suffer any symptoms or pain at the beginning showing signs only at the advanced stage. In medical diagnostic imaging systems, anomaly detection from brain images is used for cancer detection [13]. Both invasive and noninvasive approaches may be utilized to diagnose brain cancer. Biopsy is an invasive technique to extract a lesion sample for analysis. Non-invasive techniques are faster and more secure, where physical inspections of the body and brain imaging are compared. Popular non-invasive technologies to identify cancer, stroke, tumor, and other malignant elements are Computed Tomography (CT), PET (Positron Emission Tomography), Magnetic Resonance Imaging (MRI), Ultrasound, X-ray imaging [1,28,17].

Table 1 is well explained about all the types of detecting methods along with their advantages and disadvantages. The X-ray is a cheap, readily available method of tumor diagnosis, but it provides poor vision. A CT (Computed Tomography) machine produces low-resolution images and is inexpensive and simple to operate, but it also releases hazardous radiation. Ultrasound scanning is another cheap, accessible, and non-ionizing radiation-producing technique. Even though the apparatus can only generate photos with low resolution, a highly skilled professional wants to use it. The magnetic resonance imaging machine (MRI) uses powerful magnetic fields and radiofrequency pulses to assess the body's tissues and organs. It can be applied to research or diagnose various cardiac problems, cancers, accidents, etc. MRIs are slower than CT scans. One more expensive and specialized way of detecting tumors is Positron Emission Tomography (PET) [17].

**Table 1:** Tumor diagnosing techniques.

Types	Advantages	Disadvantages
CT	Fast Scanning speed, Easy to use, Sedation is not required.	Dangerous radiation, Low resolution image, Low sensitivity.
PET	Active lesion, Whole body imaging is possible.	Radiation exposure, Long acquisition time, Lower spatial resolution, More expensive, Needs specialized patient preparation
Ultrasound	Inexpensive, Quick and painless non ionizing radiation, Widely available.	Low resolution, Low sensitivity and highly experienced operator need to perform examination.
MRI	High sensitivity, image is taken in any angle, Painless, Non-ionizing radiation.	Long scan time, Expensive, Less widely available.
X-Ray	Cheaper, Easily available.	Dangerous radiation, Poor visualization.

**Fig. 4:**
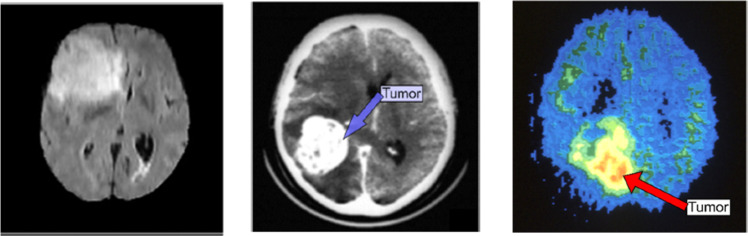
Brain tumor identification using (a) MRI, (b) CT, (c) PET [14]**.**

Head imaging systems that make use of antennas are lighter compared to recently proposed systems. The process of scanning and reconstructing images is completed in 8 s, which is much quicker than those of MRI or CT scans which are normally rated at around 10 to 30 min[18]. Brain images of these techniques are given in Fig. 4. This system is safe because it does not use ionizing radiation and operates on relatively little power, making it perfect for monitoring purposes or even instant diagnoses however, traditional MRI scanners cannot do this because they deal with static objects in bulk [19,6].

### Importance of early detection

**Fig. 5:**
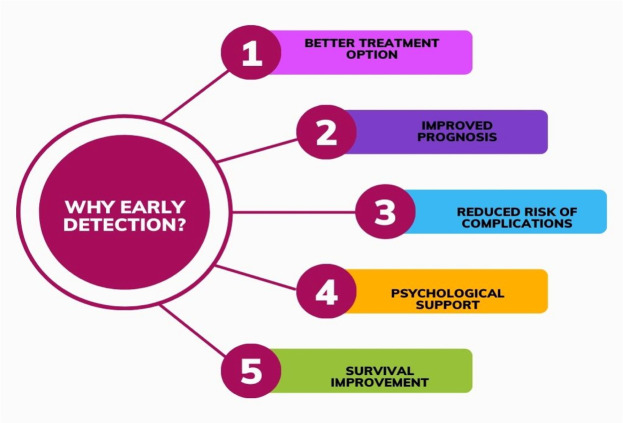
Importance of early detection.

Early detection of brain tumors offers several significant advantages [20] as shown in Fig. 5

i. Better Treatment Options- Doctors need to make a diagnosis quickly because this enables them to consider different treatment methods like surgery, radiotherapy, chemotherapy, targeted therapy, and immunotherapy. Most early-stage tumors are easier to treat and may not require aggressive interventions [21].
ii. Improved Prognosis- Detecting cancer early usually results in better outcomes because smaller tumors usually have greater chances to be completely removed and improved overall survival rates. Preventing the tumor from causing additional harm to vital brain structures is also another thing early treatment can do [22].
iii. Reduced Risk of Complications- Early detection and treatment can help control or even prevent seizures. Cognitive impairments, including memory loss, difficulty concentrating, and changes in behaviour or personality, can arise as a result of brain tumor growth and its impact on brain function. Treatment at an early stage can help prevent or manage these complications effectively.
iv. Psychological Support- Patients and their families can utilize the needed psychological and emotional support services through early detection; and enjoy effective ways of coping with their diagnosis and treatment process. Support groups make it possible for patients to start discussions with healthcare workers as they become aware in advance [23].

### Antenna technology in medical imaging

Microwave imaging (MWI) systems are presently receiving considerable attention as an option for cancer detection. In section I, it was mentioned that numerous imaging techniques exist at various microwave frequency ranges to capture the head's internal structure, tissue parameters on the head, and brain activities. X-rays, computed tomography (CT) scans, and magnetic resonance imaging (MRI) are some of the popular technologies used in head imaging treatment. Such technologies have been seen to possess some disadvantages as explained in Table 1. Although these scans have their negatives, such as ionization and hazardous radiation, which can cause cancer in the future even if taken in the recommended minimal dosages [24,18]. Microwaves are non-ionizing electromagnetic (EM) waves; they can enter human tissues without damaging health.

Phantoms act as important testing frameworks in creating medical imaging equipment due to their dielectric and electromagnetic characteristics. which closely match those of human tissues. They provide a secure and effective substitute for human testing, especially in the initial phases of verifying antenna designs, system setups, and equipment efficiency. In phantom models, a tumour-simulating area with unique dielectric characteristics can be placed at a specified location, size, and shape, enabling precise evaluation of the system's ability to detect tumours. Furthermore, phantoms facilitate system calibration and performance assessment regarding resolution, sensitivity, penetration depth, and accuracy prior to advancing to real-tissue investigations [25,26].

It has been suggested as a fast, safe, affordable, accurate high data rate system with less complexity, non-ionizing properties, portability, and extremely low power density [[24], [25], [26], [27],^28^].

**Fig. 6:**
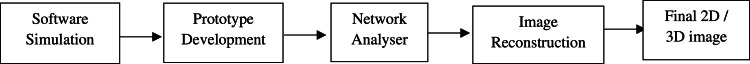
Image visualization in MWI.

Fig. 6 shows the steps to get an image visualization of a brain tumor using antenna. Initially, software simulations were conducted using software tools, followed by the creation of a prototype for initial testing to identify any parameters that may need to be adjusted using a network analyzer. Image filtering is done after this step to remove the noises. Once the results are obtained, the images are reconstructed by utilizing image reconstruction algorithms. In the end, a visual image is shown in either 2D or 3D format after the frequency domain construction is done.

**Fig. 7:**
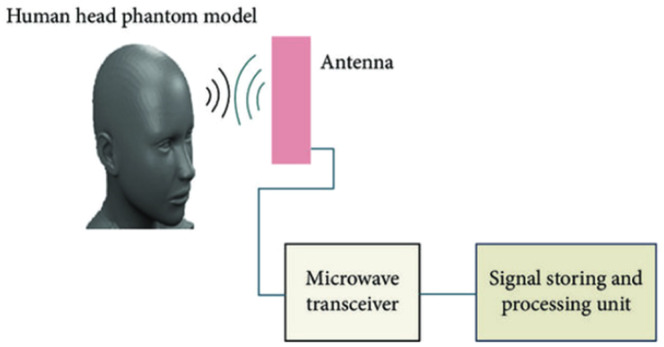
Schematic diagram microwave brain imaging system [29].

**Fig. 8:**
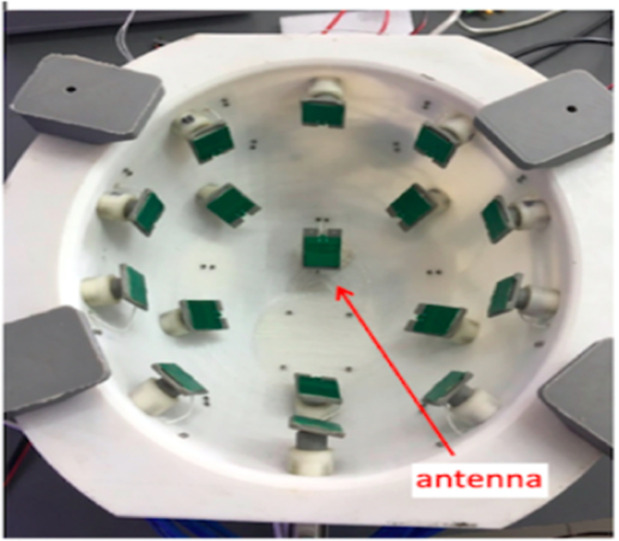
Helmet shaped head imaging system [30].

Fig. 7 shows a detailed schematic diagram of microwave imaging system and in Fig. 8 gives an idea about how an antenna array is placed on human head to visualize brain system.

### Evolution of antenna technology in medical applications

**Fig. 9:**
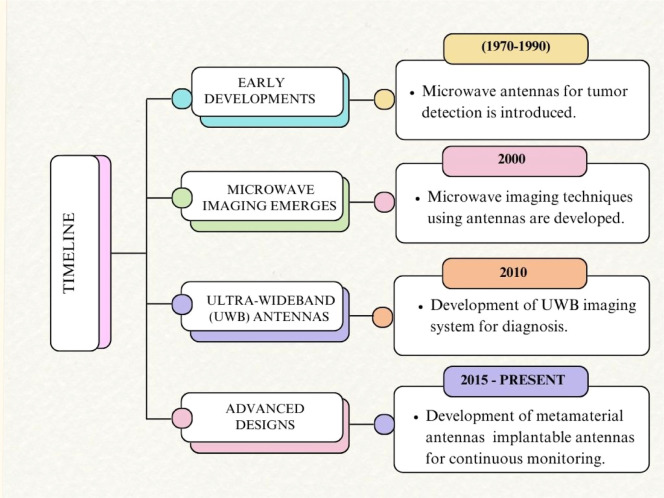
Development of antenna imaging technology.

**Fig. 10:**
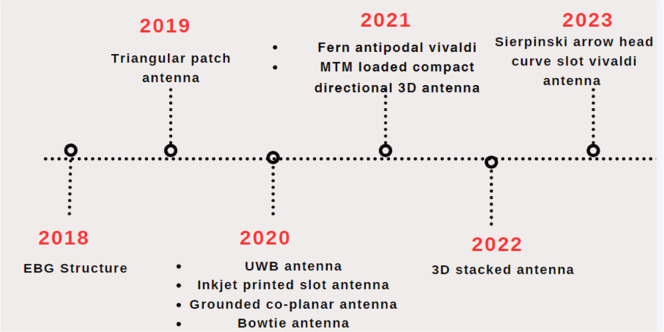
Evolution of head imaging techniques.

Microwave imaging platforms for head imaging applications use a variety of antenna types that are well-designed antennas that are smaller in size, high gain, and directivity with increased bandwidth [31]. Antenna imaging technology development in medical applications is discussed in Fig. 9. As the development of antenna technology increases, improved diagnosis, continuous monitoring, improved resolution, and enhanced sensitivity are provided. Fig. 10 shown below considers some of the antennas introduced recently to diagnose brain tumors using microwave imaging technology. Here new diagnosing techniques are discussed to overcome all the disadvantages that are mentioned in Table 1.

In 2018, an EBG structure [29] has been introduced for the early detection of tumors, which uses a flexible Roger substrate, but this antenna is not in the ISM band frequency. Later in 2019, an antenna was constructed using an FR4 substrate with an isosceles triangular patch antenna a with fractal ground plane [32], but it is not flexible for medical applications. In both cases, the size of the antenna is high, which is not suitable for medical applications. Then more designs were introduced by altering the substrates and patches [16,33,34]. However these methods do not meet the required specifications, namely frequency range, antenna size, gain, and SAR [35]. developed an antipodal Vivaldi antenna and paper designed a MTM-loaded directional antenna and a 3D stacked antenna which produces high size with SAR value.

### Role of antennas in brain tumor detection

Microwave head imaging is an emerging technology for medical diagnosis that has attracted a great deal of attention around the globe due to its cost-effectiveness, non-ionizing nature, absence of ionizing radiation, and portability. From the differences in electrical properties of the head, this technology can be used to find early cancerous tissues or hemorrhages within the human head as well as other internal abnormalities [36]. There are many requirements necessary for the right one, including but not limited to how easy it is to integrate them into one system, how small they are while maintaining their compact size, what shape they must take to remain simple in design without being too large at the same time, but they can also accommodate more features than other forms, thus ensuring that additional features are not limited simply because they take more space when placed side by side with others while boasting smaller physical dimensions, and what bandwidth is available at each point or direction of use, among others [37]. This need for multiple elements at once may be met by a microstrip patch antenna, as it has several benefits, such as low profile but wide bandwidth, gain, and directivity. An on-body FSS-backed high-gain microwave system enhances brain tumor diagnosis by improving antenna directivity, minimizing body-loading effects, and increasing penetration depth. The FSS structure reflects undesired radiation and strengthens forward waves toward the brain, leading to improved signal-to-noise ratio and more accurate tumor detection in early stages [38].

### Antenna design considerations for brain tumor detection

The Antenna is designed so that its dimensions are small enough for practical purpose [39,40]. Implantable antennas have to be physically small enough to be compatible and inserted inside the human body and, thus, researchers apply extra effort to reduce the implanted antenna size. However, antenna electrical size directly affects the electromagnetic performance where the reduction of antenna size reduces the antenna performance. Artificial Magnetic Conductor (AMC) is a special engineered surface made from metamaterials. In nature, magnetic conductors don’t really exist for radio waves, so AMC is a smart human-made trick to mimic that behavior [41,42]. The incorporation of an AMC surface improves antenna performance by increasing gain and bandwidth, reducing the antenna height, minimizing back radiation, and enhancing radiation efficiency. To better manage the complicated, nonlinear electromagnetic behavior of human head and tumor tissues, metamaterial-inspired microwave sensors with multiple detection points/bands are being used instead of a single band sensing technique [12]. MIMO antennas operate as a coordinated system where multiple elements simultaneously transmit and receive signals, rather than relying on a single antenna. This collaborative configuration enables enhanced signal processing and spatial data acquisition. In medical imaging, particularly for brain tumor detection, the improved performance of MIMO systems contributes to more accurate diagnosis and better identification of tumor characteristics [43].

Designing antennas to identify brain tumors takes into account dozens of different factors, of which some are the selection of substrate, antenna size, working range, specific absorption rate (SAR) [44], voltage standing wave ratio (VSWR), dielectric constant, gain, loss tangent, return loss, fractional bandwidth, fidelity factor, feed line, and reflection coefficient. Substrates provide the mechanical support needed to keep them stable. When the dielectric substrate becomes larger in size, the volume that an antenna occupies increases [45].

### Frequency selection

The Federal Communications Commission in America, FCC, has designated 2.4 –2.485 GHz bands for industrial, scientific, and medical (ISM) purposes [46]. Compared to lower frequencies, there are several benefits to designing antennas for biomedical applications inside the GHz range. Shorter wavelengths at GHz frequencies provide excellent precision and greater resolution for applications like near-field microwave imaging and radar-based scans. This is one of the main advantages. This enhanced resolution makes tissues more easily visible in detail and makes it possible to more precisely identify possible anomalies. Compared to lower frequencies, GHz waves provide better tissue penetration. They penetrate deep into the body with minimal attenuation making them suitable for brain imaging. It improves the capacity for diagnosis and treatment by enabling the exploration and examination of previously unreachable areas. Additionally, using GHz frequency has additional benefits, such as the ability to miniaturize which is mentioned in Table 2.

**Table 2:** Comparison table for antennas used in brain tumor detection.

SI. No	ANTENNA	SUBSTRATE	DIMENSIONS (mm)	RANGE	SAR W/kg	ε_r_	GAIN	δ	S_11_	FBW
[33]	Wideband antenna embedded with PDMS	Roger Duroid 4350B	38×24×0.168	3- 6 GHz.	–	3.48	–	0.0037	–	–
[94]	Inkjet printed slotted disc monopole antenna	PET	40 × 38×0.135	2.2–2.73GHz	0.8 (1 g)	3.2	2.78	0.022	−20	480MHz
[16]	Grounded coplanar waveguide	Rogers RO4350B	50 × 44 × 1.524	1.70–3.71 GHz	0.00233	3.66	5.65	0.0037	–	2.01 GHz
[40]	Compact unidirectional conformal antenna	PDMS	25 × 28 × 5.6	1 - 4.3 GHz.	–	–	–	–	-20	124 %
[24]	Ultra-wideband textile antenna	Plain-woven polyester Fabric	70 × 50 mm^2^	1 to 3 GHz	0.0014	2.193	2.9	0.004	–	1.198–4.055 GHz
[27]	Flexible multi-slot planar antenna array	RTV silicone rubber	28×26 mm^2^	0.6–2.5 GHz	0.5(10 g)	3.3	–	0.018	-8 dB	–
[95]	A double hollow rectangular-shaped	FR-4	0.28λ x 0.24λ x 0.006λ	1.22– 3.04 GHz	–	–	5	–	-60 dB	1.82 GHz
[35]	Fern Antipodal Vivaldi Antenna	FR-4	150×150×1.6	1.5 GHz	–	4.3	6.66	–	−10 dB	–
[96]	3D adaptive antenna array	Kapton polyimide-based substrate	5 cm x 5 cm x 0.11mm	0.5 - 1 GHz	–	3.5	7.57	0.002	- 30.44 dB	–
[62]	MTM loaded compact directional 3D antenna	Rogers RT5880	70×30×14	1.12 -2.5 GHz	0.041 W for 10 *g*	2.2	5.192	0.0009	–	76.24 %
[97]	Meander line antenna	RTV silicone rubber substrate	24 × 45 × 4	0.42–3.2 GHz	0.2 W/kg	–	–	–	–	–
[98]	K liquid crystalline polymer (LCP)	Roger ULTRALAM	7 × 7 × 0.2	2.4−2.48 GHz	–	2.9	–	–	−20.71 dB	1038.7 MHz
[63]	3D stacked	Rogers RT5880 and RO4350B	50 × 40 × 8.67	1.37–3.16	–	–	6.6 dB	0.0018	–	79.20 %
[6]	Antipodal Vivaldi antenna	FR4 dielectric substrate	–	3.2 -4.5 GHz	–	–	6.7 dB	–	–	–
[57]	Circular slot and a tiny triangular cut at the top right corner	FR4-epoxy	38×30.68 mm^2^	1.22– 3.45 GHz	–	4.4	5 dB	0.0316	–	95.71 %
[1]	Sierpinski Arrowhead Curve Slot Vivaldi Antenna	Roger RO4350B	65 × 65 × 1.6	2.35 -3.79 GHz	–	–	7.35 dB	0.0013	–	–
[99]	CPW-fed monopole antenna	PET	70 × 30 × 6	0.475 - 1.089 GHz	0.0286 W/kg	–	–	–	-10 dB	615 MHz
[12]	Rectangular patch	FR-4	12.89 × 12.89×1.50	–	–	4.3	6dB	0.025	–	–
[100]	Single-element crescent sensors	FR4	60×90 mm^2^	1–4GHz	–	4.4	–	–	–	0.8GHz

**Fig. 11:**
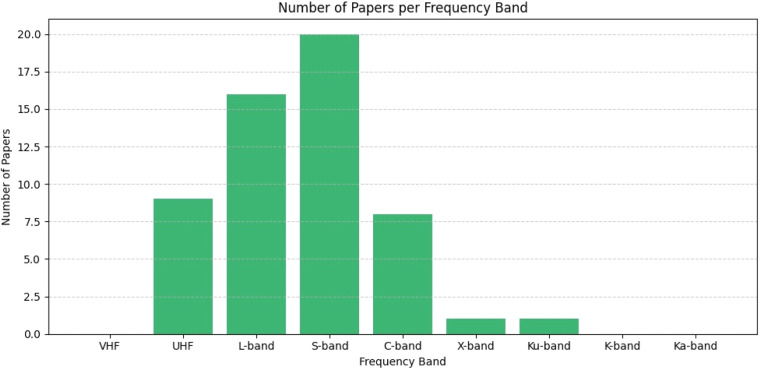
Number of papers per frequency band.

The Fig. 11 shows the frequency band selection of the papers considered. Out of 105 papers, the majority utilized the S-band range, which lies between 2 and 4 GHz. Most of the antenna uses 1 – 3 GHz frequency for tumor detection. The shorter wavelengths that characterize GHz frequencies make it possible to design tiny antennas. As a result, the possibility of miniaturization suggests that biomedical devices could be made smaller yet no less functional [31].

### Antenna size and configuration

Antenna types vary in their physical configurations and dimensions which in Table 2 [47]. An antenna size is dependent on several variables. These advantages lead to more successful and efficient methods for tumor identification and treatment.

(a) The physical dimensions of an antenna are determined by the wavelength of the signal it carries. Larger antennas are needed to effectively emit or catch electromagnetic waves at lower frequencies because of their longer wavelengths.
(b) Higher directivity antennas frequently need to be larger physically to provide the intended radiation pattern.
(c) To get better performance, large physical sizes are needed for antennas with higher gains. The size of antennas can also depend on the materials used in making them. Some scenarios prefer smaller antenna sizes for detecting brain tumors [48].

**Fig. 12:**
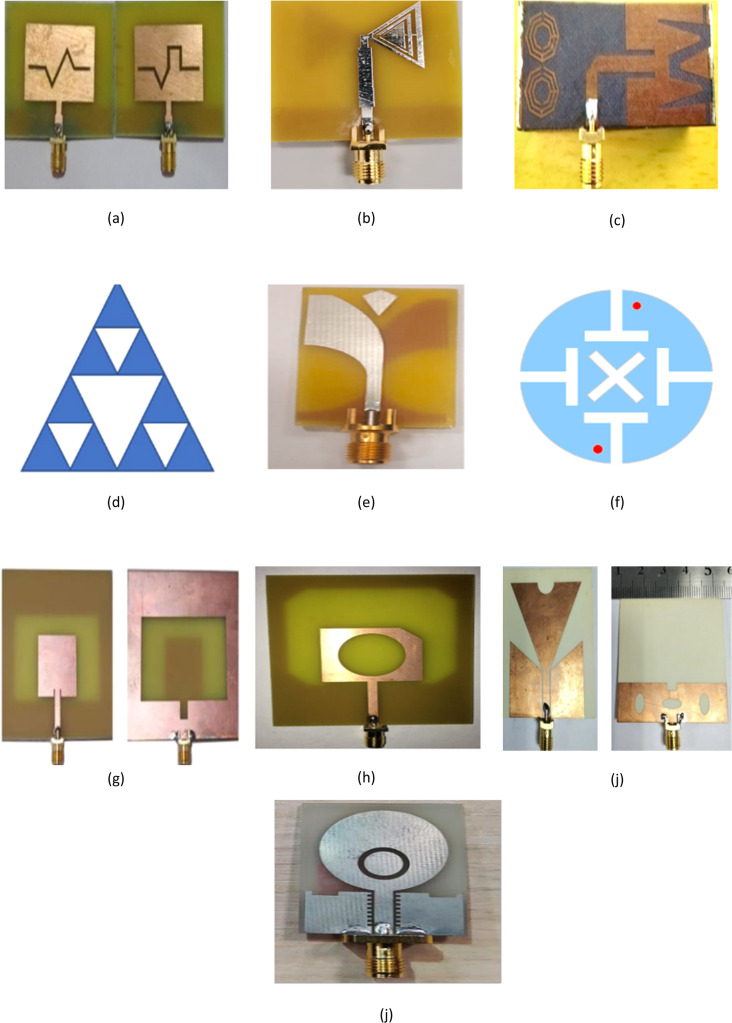
Antenna configurations (a) Fractal slot along with partial ground plane [52], (b) Bowtie antenna fabricated on FR4 substrate[53], (c) MTM-loaded 3D antenna [54], (d) Sierpinski arrowhead curve slot antenna [1], (e) Antipodal Vivaldi antenna (AVA) [6], (f) Patch with T and crossed slot [55], (g) Slotted Monopole Patch Antenna [56], (h) Circular slot in the patch and a hollow rectangular-shaped ground plane with inner slanted corners [57], (i) Slotted inverted delta- shaped wideband antenna with rectangular-shaped and elliptical-shaped slots on the ground [16], (j) Non- uniform structure on the ground, and using a split-ring resonator (SRR) on the radiation element which is placed on the FR-4 substrate [58].

Different antenna configurations are introduced to enhance gain, bandwidth and to increase surface current and these configurations are shown in Fig. 12.

A detailed analysis of various antenna configurations is tabulated in Table 3 [49].

1. **Higher resolution imaging**. Smaller antenna sizes may make it feasible to obtain finer spatial resolution, which would make it possible to identify tumors and other smaller or more detailed features within the brain tissue [50].
2. **Availability and Portability:** Smaller antennas are more useful for usage in clinical settings since they are typically more portable and manageable. They can be made to be more user-friendly and less obtrusive for both patients and medical professionals.
3. **Comfort**: Smaller antennas are able to increase the comfort experienced by the patient when they are undergoing imaging procedures. Patients having brain tumor detection scans may feel less discomfort if they are made to be more comfortable and less bulky.
4. **Cost**: smaller antennas could be less expensive to produce and maintain than bigger ones. They might also not require that much space and infrastructure, which would make them more feasible to be used in medical facilities.

**Table 3:** Detailed analysis of various antenna configurations.

Configuration Type	Variants	Frequency Range	Key Features	Disadvantages	Applications
**Microstrip Patch** [59,29,60]	Rectangular, Circular, and Elliptical patches	2–10 GHz	Compact, modifiable, Low SAR	Narrow bandwidth unless modified, Moderate gain	Phantom models
	DGS (Defected Ground Structure) and EBG (Electromagnetic Band Gap) enhanced MPAs				
	Slot-loaded or slotted patch antennas for bandwidth enhancement				
**UWB** [24,35,61]	Vivaldi antennas	3–10.6 GHz	Wide bandwidth, high resolution	More complex design and fabrication, may require careful signal processing	Confocal imaging
	Printed monopole antennas				
	Bowtie antennas				
	Planar elliptical antennas				
**Metamaterial-loaded** [62,36]	Split-ring resonators	Varies	Enhanced gain	Fabrication can be complex and expensive	Sensitive detection
	3D metamaterial structures				
**Conformal** [40]	Flexible microstrip antennas	Varies	Skin-adaptive, wearable	Mechanical stress can impact performance	On-body systems
	Polymer-based antennas				
**Antenna Arrays** [19,63]	Linear, circular, or spherical arrays	1–12 GHz	Full imaging, 3D tumor localization	High system complexity, Requires synchronization and calibration.	Clinical research

### Material selection

Various kinds of antenna arrays have been suggested and used for EM head imaging systems. In the construction of these antennas, choosing the correct substrate material is very important as it can either be conductive or non-conductive. The designed antenna must be able to be placed near a human body on the substrate that is chosen [51]. The two main materials that affect wearable antenna performance overall are conducting (radiating element) and non-conductive (substrate). Materials need to have a low relative permittivity, low dielectric loss, and a high thermal expansion coefficient to achieve increased efficiency, durability, and sufficient bandwidth. Some of the substrate material and their features are tabulated in Table 4.

**Table 4:** Types of substrate materials for MWI.

Ref. No.	Substrate	Advantages	Disadvantages
[29,64]	Roger’s R03003, RO4350B	Flexibility of high-frequency application	Thicker substrate, as higher cost, complexity, less availability
[34]	FR-4	Low cost, fabrication easy, availability	Limited high-frequency applications, not flexible
[19,33,65]	PDMS	Low cost, less reflection, flexible, durable, adjustable dielectric properties, Ease of Processing	Stiffness is less, and should be custom-made otherwise permittivity is very low and can’t reduce the size
[29,66]	Silicon Rubber	Flexible, Reduced cost, less reflection, durable, light weight, Elasticity, non-toxic, and hypoallergenic	Limited to rework ability

In every antenna, conducting materials are needed for the radiating element and the ground plane. The wearable antenna's overall performance is impacted by the non-conductive materials utilized in the design. In every antenna, conducting materials are needed for the radiating element and the ground plane. Ground planes and conductive radiating elements are required for wearable antennas to maintain proper antenna emission characteristics.

**Fig. 13:**
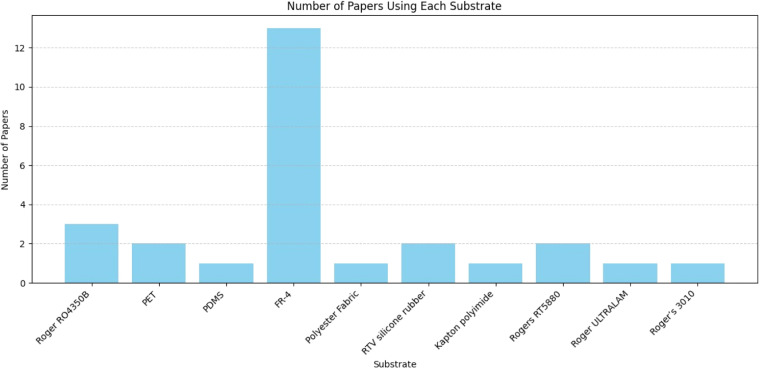
Number of substrates used.

Fig. 13 illustrates the number of papers that utilized various substrate materials for microwave imaging systems. Among the substrates considered FR-4 is the most commonly used, appearing in 13 papers, indicating its popularity likely due to its low cost and wide availability despite its higher dielectric losses. Other substrates like Roger RO4350B, Rogers RT5880, RTV silicone rubber, and PET were used in 2–3 papers each, suggesting moderate usage. Flexible and high-performance materials like Kapton polyimide, PDMS, polyester fabric, and Roger’s 3010 appeared less frequently (1–2 papers each), possibly due to higher cost, fabrication complexity, or specific application constraints.

**Fig. 14:**
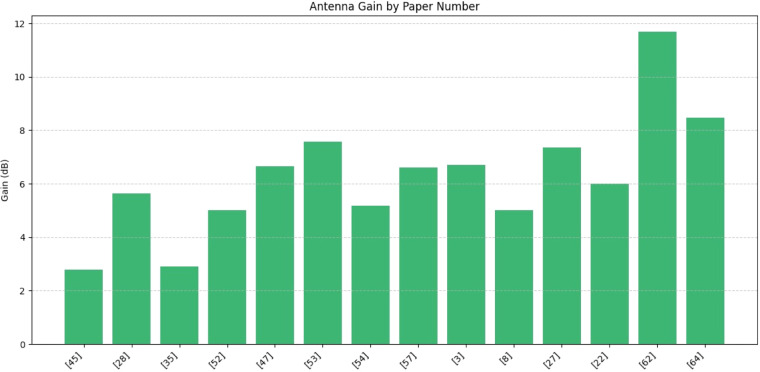
Antenna gain by paper.

Fig. 14 a wide range of gains from around 2.8 dB [45,35] to nearly 11.7 dB [62]. Papers [62,64], and [53] exhibit the highest gain values, suggesting their antenna designs are more directional or efficient. S11 (return loss) indicates how much power is reflected back to the source due to impedance mismatch. A more negative value (e.g., -60 dB) means better impedance matching and less reflected power. The best-performing paper here is [52] with S11 ≈ -60 dB, indicating excellent impedance matching. Most other papers range from around **-**10 dB to -30 dB, which is typical and acceptable for many antenna designs.

**Fig. 15:**
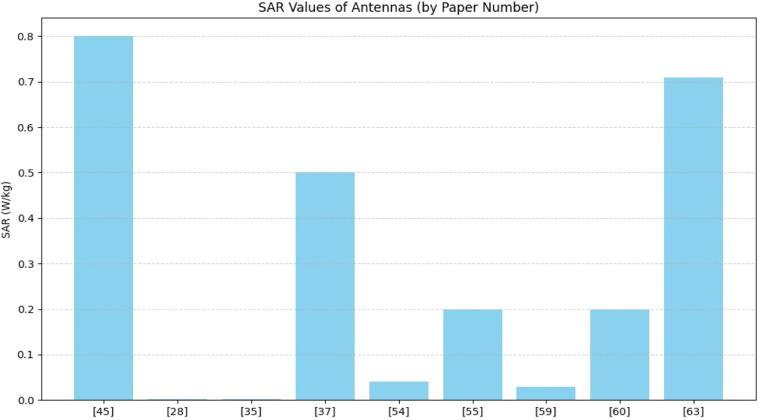
Specific Absorption Rate (SAR).

**Fig. 16:**
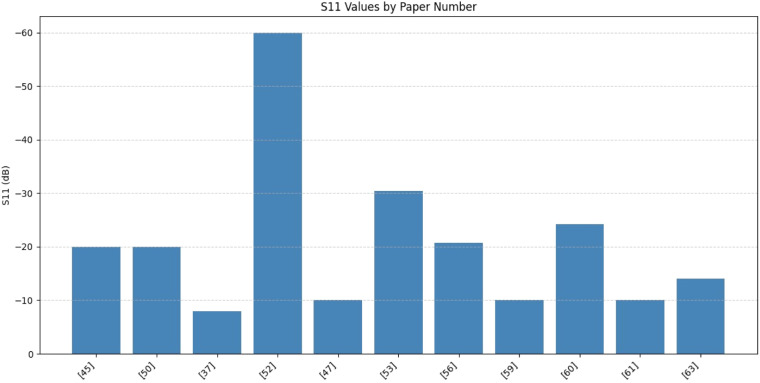
Reflection coefficient (S11).

SAR values are in Fig. 15, the lowest SAR values: [28,35,54,59] ideal for safe biomedical applications and highest SAR values: [45,63,37] acceptable but should be minimized if used on or near the human body. Papers [37,47,61] show higher (less negative) S11 values, meaning poorer performance in terms of return loss which is shown in Fig. 16. Antennas with high gain or wide bandwidth may have higher SAR, so a balance between performance and safety is crucial.

### Signal processing techniques

The term signal or image processing describes a set of operations performed on images that result in enhancement, restoration, or obtaining useful information from the imagery. Different methods and algorithms are employed in image quality improvement by noise elimination, distortion correction, and data extraction [67,68].

Image processing is a fundamental technique for enhancing image quality through noise reduction, contrast enhancement, resizing, normalization, and the application of various filters [69]. Firstly, signals are converted to digital format and saved after being picked up by antenna arrays. Next, the outcome is transformed from the time domain to the frequency domain utilizing conversion algorithms. Frequency domain signals assist in removing noise, identifying texture, edges, and patterns, and improving image contrast and details. Image preprocessing techniques such as enhancing and reducing noise are required to improve the quality and clarity of photographs of antenna components. The main goal of image enhancement techniques is to improve the interpretability and visual appeal of an image.

**Fig. 17:**
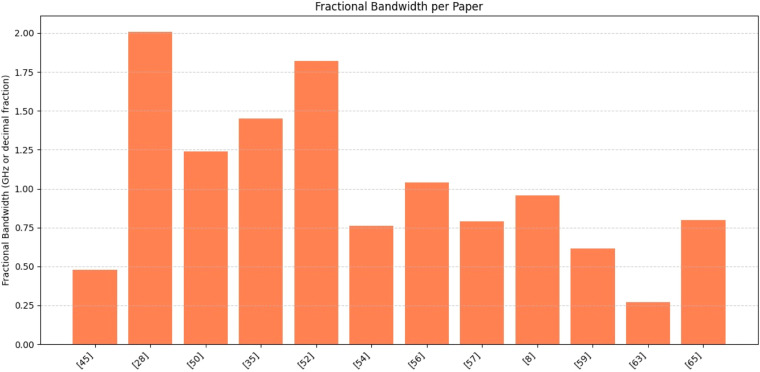
Fractional bandwidth.

Fractional bandwidth is one of the factors that determines image resolution. The values range approximately from 0.27 to 2.0, showing significant variability in design choices or technologies across papers, as shown in Fig. 17.

**Fig. 18:**
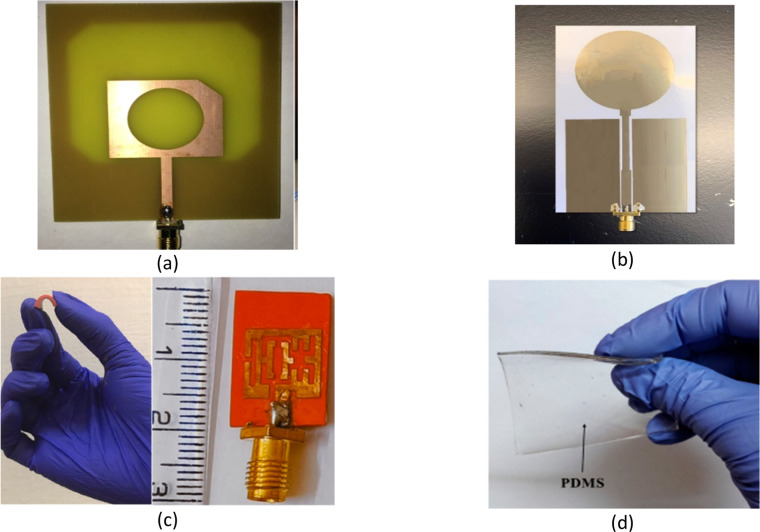
Substrate materials for head imaging antenna (a) FR4 Substrate [57], (b) PET substrate based printed pattern [70], (c) Flexible silicone rubber substrate [66], (d) PDMS substrate [65].

Fig. 18 shows different substrate materials used to design microwave head imaging antenna [59].

### Future directions and challenges

There are many techniques that are introduced for brain tumor early detection. Some of them are discussed below.

### Microwave imaging antennas

Medical imaging is the approach of imaging and visualizing organs or tissues and their functionalities for both clinical and physiological analysis. It does not only reveal the interior of a body and structures of internal organs/tissues, but it also is used for diagnosis and treatment of diseases[5]. Antenna-based microwave imaging (MWI) is an innovative method that offers a cost-effective and non-invasive approach to medical diagnosis and treatment. By utilizing electromagnetic waves in the microwave frequency range, MWI can examine the dielectric properties of biological tissues, which can provide valuable information about their overall health. One of the many applications of MWI is the detection of conditions such as breast tumors, stroke, brain injuries, skin diseases, and other ailments[31].

### Ultra-wideband antennas

**Fig. 19:**
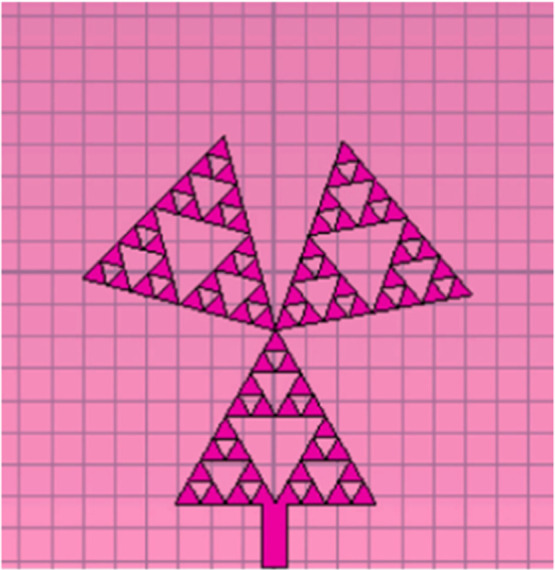
UWB fractal antennas [72].

Ultra-Wide Band (UWB) technology has gained the interest of industry and academia owing to its prospective use in performing fast data rates as well as being relatively low-powered and cost-effective[58]. Ideally, UWB technology requires a frequency band that ranges from 3.1 GHz up to 10.6 GHz for the most efficient practical performance and ideal omnidirectional radiation patterns. Fig. 19 shows an example for this UWB antenna. Ultra-wideband (UWB) antenna is high in gain, wide bandwidth, and compact in structure, and it has low power consumption compared to other microwave frequency bands [24,61,70]. By using low power levels, UWB technology lowers the risk of negative effects while also providing patient security [71].

**Fig. 20:**
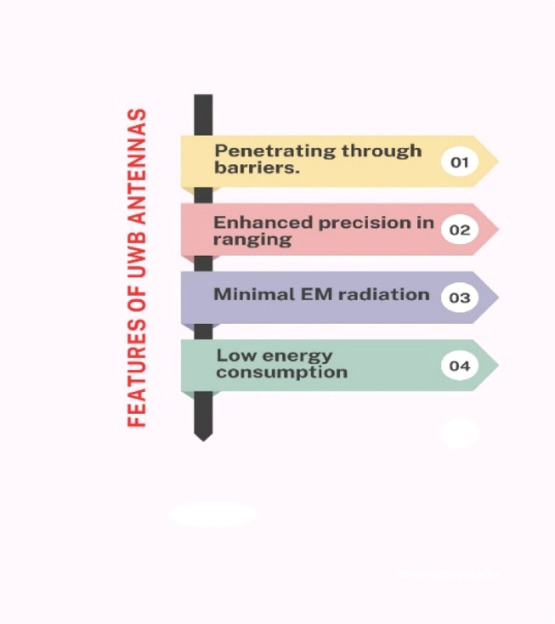
Features of UWB antennas.

The UWB characteristic is widely recognized as a significant benefit for medical engineering [73] which is shown in Fig. 20. This characteristic is beneficial in the medical field when imaging human body organs. UWB's high gain and utilization of RF pulses account for its ability to pass through walls. This allows UWB to be used in wide-area applications when obstacles are expected, while ultrasonography may also be useful in these situations. A very precise range (to the order of millimeters) is made possible by UWB without significantly interfering with narrowband systems. Ultrawideband is less electromagnetic radiation suitable especially for hospital applications. Besides, it is safe for the human body since it emits little radiation. Low energy consumption, allowing the use of battery-operated devices with extended lifespans.

### RF coils for MRI

**Fig. 21:**
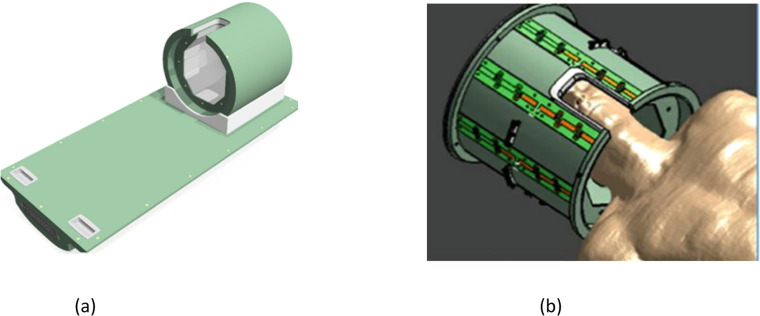
Structure of the brain coil (a) equipment, (b) RF coil placed on head [78]**.**

An essential part of magnetic resonance imaging (MRI) is radiofrequency (RF) coils, which are used to transmit the RF field to excite nuclear spins and to receive the MRI signal [74,75]. RF coils are essential for the quality of magnetic resonance imaging (MR) because they affect signal-to-noise ratio (SNR), signal homogeneity, and picture resolution. Ensuring good functioning of RF coils is crucial for maintaining consistent picture quality and preventing coil issues that could disrupt patient imaging or impair image evaluation. This is why quality control of RF coils is so important [76]. These coils are safe and flexible to use in medical imaging because they don't offer any threats to the body or health. Helmet-like coils called specialized coils for brain are used for head imaging. They are positioned above the head. All things are to be considered; MRI coils are essential to guaranteeing precise and excellent imaging for a variety of medical diagnostic applications [77]. Fig. 21 shows the structure of a RF coil which is used in MRI to improve brain imaging.

### Other emerging technologies

Current diagnosis methods face challenges such as early detection due to tumor concealment, imaging limitations, and issues of visualizing small or deep-seated tumors, difficulties in distinguishing tumor types, invasive procedures with associated risks, and the heterogeneity of brain tumors [79]. Recently, numerous researchers have been conducting studies using microwave techniques to detect brain strokes or brain hemorrhages. Nevertheless, these microwave systems have additional antenna units for microwave imaging (MWI) of human heads [37]. Because multiple antennas are used, the overall setup becomes complicated and large in size. In order to enhance precision and efficiency and allow for live tracking of health issues and vital indicators in the field of biomedicine, numerous artificial intelligence (AI) methods have been combined with microwave technology [31]. Early brain tumor detection is essential for effective treatment. various novel technologies are under development to enhance the identification of brain tumors, such as:

#### Artificial intelligence and machine learning and deep learning brain tumor detection

**Fig. 22:**
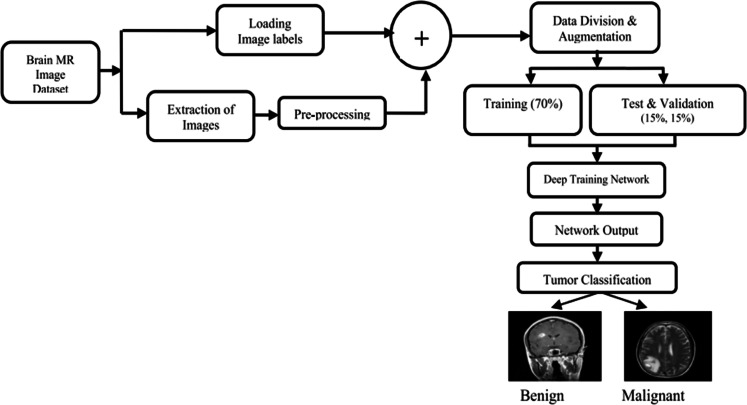
AI and ML and Deep learning brain tumor detection [86]**.**

Fig. 22 gives an overview of AI and ML and Deep learning brain tumor detection steps. Artificial intelligence (AI) has been developed to help in the identification and treatment of complicated maladies that attack the brain like tumors through integration of technologies like big data analytics, machine learning, and deep learning. In order to analyze brain imaging techniques like Magnetic Resonance Imaging (MRI), it can also identify and classify tumors. When it is used to complement other diagnostic tools which rely on human skills alone, AI algorithms may further aid in deciding on the extent or class or location or type of aggressiveness of a tumor [80]. This way, they make possible to make more precise diagnostic and treatment decisions during examination of a patient since this technology reduces chances [81,82].

Machine learning (ML) is just a portion of artificial intelligence (AI). On a large amount of data, finding patterns and correlation is trained on the algorithms to respond to various problems better among which best prediction is reached at [83]. In this manner, ML systems are improved gradually, they become more effective and one can depend on them as they handle a given task [84]. Also nowadays many people use deep learning methods during medical imaging applications [54]. Deep learning happens to be the critical component of machine learning and its foundation lies in complex artificial neural networks. This depth arises out of an artificial neural network structure that comprises multiple input/output and hidden layers often connected among them. In comparison to traditional machine learning algorithms, deep networks give significantly improved outcomes when it comes to comprehension; they can recognize objects or voices alike with greater efficiency as well predict future occurrences for medical information processing [85].

#### Liquid biopsy

A non-invasive procedure called liquid biopsy, where blood samples are examined for biomarkers unique to tumors, such as extracellular vesicles and circulating tumor Deoxyribonucleic Acid (DNA). This technique can provide useful information on the tumor's genetic composition as well as follow the tumor's evolution over time [87,88].

**Fig. 23:**
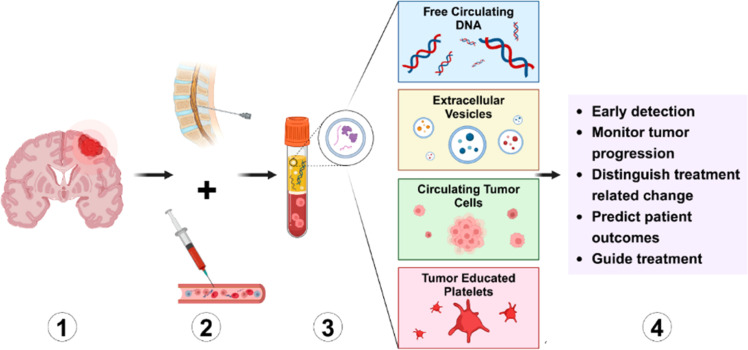
Overview of liquid biopsy [22]**.**

Fig. 23 explains about the procedure of liquid biopsy:

(1) Tumor Source: Tumors located in the central nervous system (CNS) release molecular and cellular constituents into adjacent biological fluids, including blood and cerebrospinal fluid (CSF). These materials are then emitted into circulation.
(2) Sample Acquisition: Liquid biopsy entails gathering patient blood or cerebrospinal fluid via less invasive methods. This enables the extraction of tumor-derived biomarkers without the need for direct tissue sampling.
(3) Essential Biomarkers: Free Circulating DNA (cfDNA): Cancer cells send DNA fragments into the blood, which contain specific mutations or methylation signatures associated with the tumor.

• Extracellular Vesicles (EVs): Cancer cells emit nanoparticles that carry proteins, RNA, and DNA, which mirror tumor biology and development.
• Circulating Tumor Cells (CTCs): Whole tumor cells that leave the primary tumor and enter the bloodstream, offering direct cellular insights into the cancer.
• Tumor-Educated Platelets (TEPs): Tumor cells reprogram platelets to exhibit tumor-specific RNA and protein markers, facilitating tumor identification and analysis.

(4) Clinical Applications: The elements of liquid biopsy have various diagnostic and prognostic applications, such as:

• Early Detection: Spotting tumors prior to the onset of clinical symptoms. Observing Tumor Development: Following alterations in tumor biology as time progresses.
• Identifying Treatment-Related Changes: Distinguishing real tumor progression from pseudo progression or radiation necrosis.
• Forecasting Patient Results: Evaluating tumor severity and possible treatment reactions.
• Guiding Therapy: Tailoring individualized treatment plans through continuous tumor observation.

#### Optical coherence tomography (OCT)

**Fig. 24:**
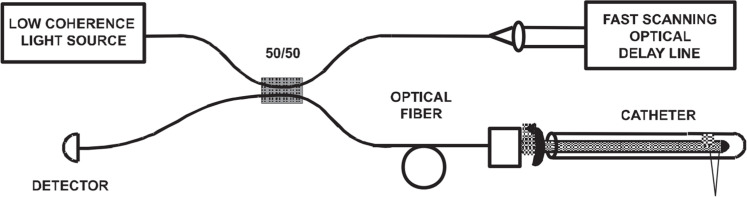
Schematic of an OCT system [91]**.**

OCT can be readily incorporated into a surgical workflow and enables noninvasive, contactless in vivo application when combined with a surgical microscope [89]. It uses optical near-infrared light and low-coherence interferometry to visualize the biological tissue microstructure. When a brain tumor is detected, optical coherence tomography (OCT) shown in Fig. 24 can help identify the precise location of the tumor by distinguishing between healthy and tumorous tissues based on differences in their optical characteristics [90].

### Challenges

Many technical and clinical difficulties arise in the identification of brain tumors using antennas. Common challenges in detection include the (i) Generated pictures that are distorted, hazy and lack clarity, (ii) It is difficult for a non-expert radiologist to identify the tumor and its location, (iii) Data Processing, Safety Concerns and Clinical validation [54,22]. As compared to MRI and CT scans, EM imaging technique is not able to offer full information about structural organs within the body [3].

### Technical challenges

Numerous difficulties arise in the midst of MWI. Optimizing antenna size, shape, and material for efficient microwave radiation presents some hardware challenges [92]. Furthermore, there is signal weakening during testing, signal reduction caused by tissue absorption, and scattering, as well as minimizing electromagnetic interference from external sources. Improving sensor positioning for precise image reconstruction and ensuring precise system calibration for dependable image creation. Challenges in signal processing during image reconstruction include developing strong algorithms, effective noise reduction, removing artifacts, interpreting data, achieving real-time processing, and analysis. The most suitable frequency for the MWI system to sense tissue is still under study, with no agreement on the best bandwidth for MWI yet [93]. When designing microwave imaging systems, it is crucial to consider their physical compatibility with examining the human body. Designing a full system with appropriate sensors and creating target detection may be necessary. Microwave radiation can penetrate the human body and gather structural and functional information about tissues by analyzing scattered signals. Some of the important challenges are discussed here.

Microwaves need to penetrate the skull and brain tissues successfully. The dense and diverse nature of these tissues can significantly reduce the effectiveness of the microwaves, resulting in a loss of signal strength from the inner regions of the brain. The skin, skull, CSF, and brain matter surrounding the head have varying dielectric properties, causing signal attenuation and scattering, making signal interpretation challenging. Under these circumstances, making a clear differentiation between the healthy and the tumor tissues is not an easy task. Noise from the environment or other electrical sources can corrupt the microwave signals thus imposing the need for advanced noise suppression and interference techniques. Accurate tumor localization and characterization in the brain requires advanced imaging and signal processing techniques due to the need for high resolution. Creating antenna arrays that ensure complete brain coverage without compromising signal quality is a challenging task. This includes maximizing the efficiency of the antennas by adjusting their size, shape, and layout to achieve optimal results.

### Clinical challenges

Developing compact, efficient antennas suitable for clinical applications. Many clinical challenges are faced during tumor detection. Some of them are discussed here.

• Patient related challenges: Changes in tissue electrical characteristics impact the quality of images. The accuracy of the image is reduced by motion artifacts caused by patient movement. Following is the skull thickness, which impacts image quality as signal attenuation is caused by skull thickness. The structure of the brain is a broad factor that makes interpreting images difficult. In conclusion, characteristics of the tumor.
• Imaging related challenges: Offer restricted spatial resolution and precision. Creating strong algorithms to accurately reconstruct images. Maximize signal-to-noise ratio to ensure dependable image creation. Accomplish real-time processing and analysis of images.
• Safety and comfort challenges: Ensure all processes are done safe and within required limits, especially Magnetic Resonance Imaging (MRI). Limit patient discomfort during these imaging techniques. Limit exposure to radiations. Make sure that control of infections is done.
• Clinical validation challenges: Perform clinical studies to prove the effectiveness of the system. Seek permission from the authorities for the system to be used on patients. Imaging protocols must be made uniform. Efforts should be made to reduce the differences among various operators of the same machine.

## Conclusion

This review discussed various microwave head imaging methods for the early detection of brain tumors. Antenna-based method is an advanced, non-invasive diagnostic methodology. These systems leverage the dielectric properties of brain tissues to detect anomalies indicative of tumors, offering advantages such as portability, reduced costs, and real-time results compared to conventional imaging techniques like X-ray, ultrasound, MRI, and CT scans. This review analyzed different substrate materials suitable for human tissue and antenna configurations to enhance the performances. Flexible and customized substrates are used to achieve higher performance in terms of SAR, gain, frequency, and bandwidth. Circular and elliptical slots on the ground and patches are also considered to improve performance. Metamaterials added to the bottom layer also show promising results. Different imaging techniques are also discussed, which are used to obtain high-resolution images. Other emerging technologies for early detection are also discussed.

## Ethics statements

The platform(s)’ data redistribution policies were complied with.

## Supplementary material *and/or* additional information [OPTIONAL]

None.

## CRediT authorship contribution statement

**Jinu Mathew:** Investigation, Writing – original draft. **Osamah Ibrahim Khalaf:** Conceptualization, Methodology. **Navin M George:** Supervision, Formal analysis. **Ancy Michel:** Data curation, Writing – review & editing. **Neethan Elizabeth Abraham:** Resources, Formal analysis. **Deema Mohammed Alsekait:** Project administration, Funding acquisition. **Sharf Alzu’bi:** Data curation, Writing – original draft. **Diaa Salama AbdElminaam:** Writing – review & editing.

## Declaration of competing interest

The authors declare that they have no known competing financial interests or personal relationships that could have appeared to influence the work reported in this paper.
